# Mycelial morphology influenced aerobic DNRA in *Streptomyces mediolani* EM-B2: short rod-shaped mycelium showed markedly greater efficiency than long filamentous mycelium

**DOI:** 10.1128/spectrum.00709-26

**Published:** 2026-07-02

**Authors:** Manman Zhang, Tengxia He, Cerong Wang, Yixiao Liao, Hong Zhao, ChengTao Jin

**Affiliations:** 1 Key Laboratory of Plant Resource Conservation and Germplasm Innovation in Mountainous Region (Ministry of Education), Guizhou Key Laboratory of Agricultural Microbiology, College of Life Sciences, Guizhou University 71206, Guiyang, Guizhou Province, China; 2 Key Laboratory of Karst Georesources and Environment (Guizhou University), Ministry of Education, Guizhou University 71206, Guiyang, Guizhou Province, China; Institute of Microbiology, Chinese Academy of Sciences, Beijing, China; Beijing Normal University, Beijing, China

**Keywords:** aerobic DNRA, different morphology, high ammonium production, nitrite reductase, ^15^N isotope tracing

## Abstract

Microbial dissimilatory nitrate reduction to ammonium (DNRA) metabolism offers a novel eco-friendly ammonium production pathway. However, most reported anaerobic DNRA consortia showed low nitrite/oxygen tolerance, limited ammonium yield, and scarce reported pure-cultured DNRA functional actinomycete. This work revealed an aerobic morphology-dependent DNRA strain of *Streptomyces mediolani* EM-B2 with glucose as a carbon source. In terms of DNRA efficiency, the short rod-shaped mycelium was markedly superior, with 75.82% of the converted nitrate recovered as ammonium (63.61 mg/L) compared to only 46.61% (44.78 mg/L) for the long filamentous mycelium. To date, no comparable studies have reported such morphology-dependent metabolic differences in DNRA in actinomycetes. The strain EM-B2 could tolerate and utilize 300 mg/L of nitrite to produce 75.46 mg/L of ammonium nitrogen. Furthermore, nitrite reductase (NIR) played a key role in DNRA catalysis, with an enzyme specific activity of 0.024 U/mg protein. Combined analysis of metabolite and ¹⁵N-tracing experiments confirmed ammonium production primarily via the pathway of NO₃⁻ → NO₂⁻ → NH₄^+^. This study provided a high-performance microbial agent and a technical strategy for mitigating nitrogen loss while enhancing soil nitrogen retention/recovery.

**IMPORTANCE:**

In terms of DNRA efficiency, the short aerial hypha was markedly superior, with 75.82% of the converted nitrate recovered as ammonium (63.61 mg/L) compared to only 46.61% (44.78 mg/L) for the long filamentous mycelium. To date, no comparable studies have reported such morphology-dependent metabolic differences in DNRA in actinomycetes.

## INTRODUCTION

Ammonia is a key compound widely used in fertilizers and chemical feedstocks, while also emerging as a promising zero-carbon energy carrier. To date, ammonia has become extensively integrated into human activities and has been widely released into ecosystems. Especially in agriculture, ammonium fertilizer is easily retained in the soil due to its electrostatic affinity with soil components, thus being absorbed and utilized by plants. This feature makes ammonium fertilizer the first choice for nitrogen-loving crops (rice) (1). Previous reports indicated that ammonium fertilizers were accounted for 80% of the total nitrogen fertilizers in cropping systems (2, 3). However, approximately half of the global ammonium fertilizer supply is currently synthesized via the highly energy-intensive Haber-Bosch process, which operates under high-temperature (400℃–500℃) and high-pressure (150–350 atm) conditions, with iron- or rhenium-based catalysts (4, 5). This process consumes 2% of the global energy supply annually and emits 450 million tons of carbon dioxide, severely disrupting the nitrogen cycle and ecological balance (6). Consequently, there is an urgent demand for actively developing greener, more efficient, and sustainable alternative technologies, which can efficiently produce ammonium and fulfill the requirements of energy conservation and environmental protection.

Dissimilatory nitrate reduction to ammonium (DNRA) is a key microbial pathway in the nitrogen cycle that diverts nitrate from nitrogen loss toward ammonium retention. This process plays a significant role in reducing nitrogen leaching in agricultural soils and nitrous oxide (N₂O) emissions from ecosystems. Currently, most of the reports on DNRA indicate that bacteria perform this process under anaerobic conditions, as exemplified by *Pseudomonas* sp. DKF (18.11% of ammonium production) (3) and *Desulfovibrio* sp. CMX (60% of ammonium production) (7). However, several bacterial strains have the ability to perform DNRA under aerobic conditions, which exhibit low nitrate conversion efficiency and limited ammonium production. For instance, *Pseudomonas putida* Y-9 converted 88.47% of nitrate (from 116.63 to 13.45 mg/L) after 36 h of aerobic cultivation; however, the DNRA pathway contributed only 3.42 mg/L of ammonium (8). Meanwhile, nitrite is a crucial intermediate in DNRA but exerts toxic effects on most of the bacteria. Consequently, exogenous additives are generally required for these bacteria to achieve or enhance DNRA efficiency when utilizing nitrite, require exogenous additives to perform, or enhance DNRA efficiency when utilizing nitrite. For example, Su et al. (9) observed a strong inhibitory effect on DNRA in *Escherichia coli* without ascorbic acid, whereas adding 5–10 g/L ascorbic acid increased nitrite to ammonium conversion efficiency to >89% (89.23%–89.59%). However, such additive supplementation increased operational costs and risks of secondary contamination. Consequently, there is an urgent need to discover novel microorganisms capable of efficient aerobic DNRA while tolerating and utilizing high-concentration nitrite for high-level ammonium production. Until today, DNRA processes have been extensively studied in *Thiobacillus* and *Escherichia*, involving pathways including NO₃⁻→NO₂⁻→NH₄^+^, NO₃⁻→NO₂⁻→NH₂OH→NH₄^+^, and NO₃⁻→NO₂⁻→NO→NH₄^+^ (3). Nevertheless, the functional diversity of actinomycetes, particularly *Streptomyces*, in DNRA-driven nitrogen retention remains largely unexplored.

*Streptomyces* has attracted considerable research interest owing to its remarkable metabolic versatility and environmental adaptability. Studies have shown that the morphology of *Streptomyces* has a significant impact on the yield and variety of secondary metabolites. For instance, Zhao et al. (10), through gene overexpression experiments, confirmed that the accelerated formation of *Streptomyces coelicolor* M145 aerial mycelium would result in the production of intracellular and extracellular actinorhodin in 5 L fermenters being six times and twice as high as that of the original strain, respectively. Koebsch et al. (11) demonstrated that the production of undecylcarbendazim in *Streptomyces penicillium* significantly increased with the growth of mycelial bundles. However, the influence of different mycelial forms of *Streptomyces* on its aerobic DNRA has not been studied. *Streptomyces mediolani* EM-B2 has been reported to exhibit exceptional heterotrophic nitrification-aerobic denitrification and preliminary aerobic DNRA capabilities ( 12, 13). Notably, this strain EM-B2 demonstrates remarkable morphological plasticity (long filamentous mycelium and short rod-shaped mycelium), a differentiation that may profoundly influence its metabolic traits. Nevertheless, the association between mycelial morphology and DNRA functionality has never been investigated. Therefore, the connection between the mycelial morphology type of strain EM-B2 and the function of DNRA is worth exploring. Furthermore, although most known DNRA strains are sensitive to high nitrite concentrations, the ability of *Streptomyces* to perform DNRA under elevated nitrite stress (>100 mg/L, typical in fertilized soils or wastewater) remains unknown. The lack of data on key enzymes (nitrite reductase, NIR) and isotopic verification further impedes the understanding of DNRA mechanisms in this ecologically significant genus. Collectively, investigating the DNRA capacity and pathways of strain EM-B2 under different morphologies and high-nitrite conditions will provide superior microbial resources for soil nitrogen retention.

This study presents the first comprehensive investigation into the morphology-dependent DNRA capability of *S. mediolani* EM-B2. It systematically evaluated the following: (i) the difference in DNRA efficiency between the long filamentous mycelium and short rod-shaped mycelium morphologies of strain EM-B2 under aerobic conditions; (ii) DNRA capacity under low-concentration nitrate/nitrite conditions; (iii) ammonium production kinetics under high nitrite stress (up to 500 mg/L); (iv) the catalytic role of NIR revealed through enzyme activity assays; and (v) the DNRA pathway based on ^15^N isotopic-tracing and nitrogen metabolite analyses. This work not only revealed the superior DNRA performance of the short rod-shaped mycelial morphology but also demonstrated its unprecedented nitrite tolerance (up to 300 mg/L) coupled with high ammonium production. This study provides a novel microbial resource and mechanistic insights for enhancing nitrogen retention efficiency in both engineered and natural environments.

## MATERIALS AND METHODS

### Medium and synthetic wastewater

Lysogeny broth (LB, pH = 7.2) containing 10.0 g/L of NaCl, 5.0 g/L of yeast extract, and 10.0 g/L of tryptone was used for activation and expansion culture.

DNRA synthetic medium (pH = 7.2) containing 0.72 g/L of KNO_3_, 0.01 g/L of Fe_2_(SO_4_)_3_, 0.01 g/L of CaCl_2_, 0.04 g/L of MgSO_4_, 3.75 g/L of C_6_H_12_O_6_, 1.50 g/L of KH_2_PO_4_, and 3.50 g/L of K_2_HPO_4_ was applied to detect the DNRA ability of strain EM-B2.

DNRA medium with nitrite as the nitrogen source contained 0.49 g/L of NaNO,_2,_ 0.04 g/L of MgSO_4_, 1.50 g/L of KH_2_PO,_4,_ 3.75 g/L of C_6_H_12_O_6_, 0.01 g/L of CaCl_2_, 3.50 g/L of K_2_HPO_4_, and 0.01 g/L of Fe_2_(SO_4_)_3_, pH = 7.2.

DNRA medium with low concentrations of nitrate and nitrite contained 0.10 g/L of NaNO_2_/KNO_3_, 3.75 g/L of C_6_H_12_O_6_, 0.01 g/L of Fe_2_(SO_4_)_3_, 0.04 g/L of MgSO_4_, 1.50 g/L of KH_2_PO_4_, and 3.50 g/L of K_2_HPO_4_, pH = 7.2.

The LB and synthetic wastewater were sterilized for 30 min at 0.11 MPa and 121°C. All chemicals were of analytical grade.

### Bacterium

*S. mediolani* EM-B2 was obtained from previous laboratory isolation (12). The strain EM-B2 was stored in a 30% glycerol solution at −80°C.

### Identification of strain EM-B2 of different morphologies

The two morphologies of strain EM-B2, namely long filamentous mycelium and short rod-shaped mycelium, arise spontaneously during routine cultivation, with the short rod-shaped form deriving from the long filamentous one during cultivation. Both morphotypes were separately preserved and subjected to over 10 successive subcultures. No morphological reversion occurred under any tested temperature, confirming that each form remains stable once established. The strain EM-B2 with different morphologies, previously stored at −80°C, was separately inoculated into liquid LB medium. The cultures were then activated by incubation in a shaker incubator at 28°C and 150 rpm for 36 h. Subsequently, to avoid damaging the mycelium of actinomycete strain EM-B2, the supernatant was carefully removed by gravity filtration using a funnel. Finally, the remaining biomass was carefully transferred into an electron microscopy preservation solution and sent to Seville Biotech Co., Ltd. (Wuhan, China) for scanning electron microscopy (SEM) observation.

### Detection of DNRA ability of strain EM-B2 in different morphologies

*S. mediolani* EM-B2 of different morphologies were activated and expanded according to the aforementioned method and were centrifuged at 7,000 rpm for 5 min. Subsequently, the bacterial mycelia were washed three times by repeated centrifugation using 40 mL of sterile water each time. After washing, the mycelium was inoculated at a concentration of 3.85 g/L into a simulated wastewater medium. This medium contained glucose as the carbon source and either potassium nitrate or sodium nitrite as the sole nitrogen source. All experiments, including an uninoculated control treatment, were set up in triplicate. The cultivation was conducted for 216 h under conditions of 28°C and 150 rpm. After the cultivation period, the concentrations of each inorganic nitrogen species (nitrate, ammonium, nitrite, and hydroxylamine) were determined according to the method reported by He et al. (14). The inorganic nitrogen transformation efficiency (En) and ammonium production efficiency (Ef) were calculated using the following formulas:


(1) $$En=\frac{C_{1}-C_{2}}{C_{1}}\times 100$$

(2) $$Ef=\frac{C_{3}}{C_{1}-C_{2}}\times 100$$

where C₁, C₂, and C₃ represent the initial inorganic nitrogen concentration, final inorganic nitrogen concentration, and final ammonium concentration, respectively. Concurrently, the pH value, fresh weight, and dry weight of the samples were also measured.

### Assessment of strain EM-B2 for reducing high concentrations of nitrite to ammonium

Strain EM-B2 was activated, expanded, and washed as described above. It was then inoculated at a concentration of 3.85 g/L into media supplemented with high-concentration nitrite at gradient levels (100–500 mg/L). Subsequently, the cultures were incubated in a shaker at 28°C and 150 rpm. After the cultivation period, the culture broth was collected for the determination of various inorganic nitrogen indices. A blank control, consisting of the same medium without inoculation of strain EM-B2, was included, and all treatments were set up in triplicate.

### Specific activity detection of nitrite reductase

Following activation and washing as described in Section 2.4, strain EM-B2 was inoculated at 3.85 g/L into the wastewater medium and then incubated with shaking at 28°C and 150 rpm for 216 h. After cultivation, samples were collected and subjected to ultrasonication for 3 min using a Scientz-IID ultrasonic homogenizer (300 W power, 3 s on/7 s off cycle). The culture suspension was centrifuged at 10,000 × *g* for 10 min, and the supernatant was collected as the crude enzyme extract. The protein concentration of strain EM-B2 was quantified using a BCA protein assay kit (Solarbio, Beijing, China). The activity of nitrite reductase was determined using a nitrite reductase activity assay kit (COMIN, Suzhou, China), with nitrite content serving as the indicator of NIR activity; a control reaction mixture without enzyme extract was included. Specific enzyme activity (U/mg) was calculated as the ratio of the amount of enzyme required to convert 1 µmol of substrate per minute to the protein concentration in the assay system.

### ^15^N isotope tracer

Following activation and washing as described above, the suspended mycelia of strain EM-B2 were inoculated at a wet weight of 3.85 g/L into 300 mL of sterile synthetic wastewater medium (contained within a 1 L vessel) amended with 10 atom% Na¹⁵NO₃. After thorough mixing post-inoculation, 60 mL aliquots of the medium were aseptically transferred into two separate sterile 100 mL conical flasks. The remaining medium was then incubated at 28°C and 150 rpm. The withdrawn 60 mL samples were centrifuged at 6,500 rpm for 5 min; the bacterial sediment was discarded, and the supernatants were transferred into clean 100 mL conical flasks and stored at −20°C. The other withdrawn 60 mL sample was washed and then subjected to ultrasonication (5 s pulse on/6 s pulse off, 400 W power, and a total duration of 15 min). The ultrasonicated suspension was centrifuged at 10,000 rpm, and the supernatant was transferred to a clean 100 mL conical flask and also stored at −20°C. An uninoculated simulated wastewater medium served as the control treatment, with all experimental groups set up in triplicate. After 216 h of cultivation, the aforementioned sampling and processing steps were repeated. The concentrations of nitrate and ammonium, pH, fresh weight, and dry weight were determined. The processed samples were subsequently sent to Nanjing Normal University for ¹⁵N isotope detection.

### Analytical methods

The Microsoft Excel 2016, SPSS Statistics 27, and Origin 2022 software were used for data calculation, statistical significance analysis, and line chart generation. Biorender website was applied to create graphics. All the results were presented as means ± standard deviation for three replicates.

## RESULTS AND DISCUSSION

### The different mycelial morphologies of strain EM-B2

During routine cultivation, strain EM-B2 spontaneously gave rise to two stable morphologies (long filamentous mycelium and short rod-shaped mycelium, with the latter originating from the former during growth). Both morphotypes remained stable after more than 10 successive subcultures under all tested temperatures, with no reversion observed. However, the aerobic DNRA capabilities of the two stable morphotypes derived from the same original isolate strain EM-B2 are unclear. To investigate this phenomenon, SEM was conducted first in this study to enable subsequent comparison of the DNRA capability and differences between the two morphologies of strain EM-B2.

**Fig 1:**
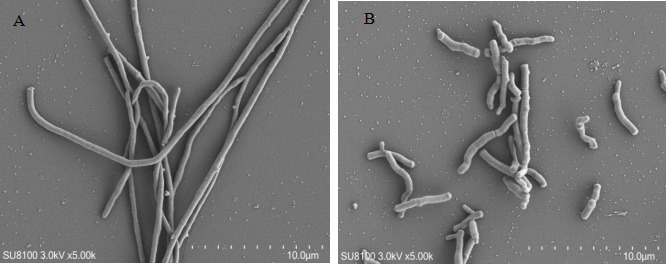
Cell morphology under scanning electron microscope (5,000×). (**A**) Long filamentous mycelium. (**B**) Short rod-shaped mycelium.

SEM observations revealed that the long filamentous mycelium of strain EM-B2 displayed long branching filaments exceeding 200 μm in length (Fig. 1A). In contrast, short rod-shaped mycelia were rod-like and branched, ranging from 24 to 56 μm in length (Fig. 1B). The mycelial morphology of actinomycetes was influenced by genetic regulation. Mycelial morphology was controlled by genes encoding glycosylated proteins associated with the cell surface. These glycans mediated the aggregation between spores and hyphae, leading to the formation of granular mycelial clusters originating from different individuals (15). Previous studies have identified that genes such as *clsA*, *glxA*, and *dtpA* were involved in the formation of the hyphal structure in *Streptomyces lividans* (16–18). The proteins encoded by these genes participate in the synthesis of extracellular glycans, thereby regulating the formation of mycelial pellets. If glycan synthesis was impaired, the mycelium exhibited a dispersed structure (19). Extracellular glycans played a crucial role in the formation of *Streptomyces* pellets, although the underlying mechanism remained unclear. Therefore, the morphological differentiation of strain EM-B2 was likely associated with the expression of its relevant genes and the synthesis of extracellular glycans. However, whether these two morphotypes arise from phenotypic plasticity (same genotype) or spontaneous genetic mutation (different genotypes) remains to be determined. In the present study, we therefore refer to them as two stable morphotypes derived from the same original isolate, rather than asserting genetic identity. Therefore, it is necessary to conduct further research on the DNRA ability of this strain EM-B2 at the metabolic and molecular levels in different forms.

### Analysis of DNRA capabilities of strains EM-B2 in different morphologies

#### DNRA with nitrate as the nitrogen source

**Fig 2:**
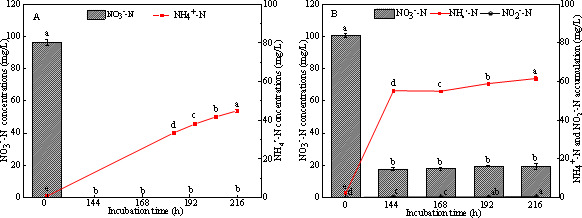
Effects of EM-B2 strains with different morphologies on DNRA using NO_3_^-^-N as nitrogen source. (**A**) The NO_3_^-^-N transformation and NH_4_^+^-N accumulation performances of the long filamentous mycelium strain EM-B2. (**B**) The NO_3_^-^-N transformation, NH_4_^+^-N and NO_2_^-^-N accumulation performances of the short rod-shaped mycelium strain EM-B2. Values are means ± SD for three replicates. Different letters indicate significant differences between treatments at *P* < 0.05.

SEM revealed significant morphological differences between the two forms of strain EM-B2. However, the effects of these morphological variations on the DNRA process of strain EM-B2 remained unclear. Therefore, this study investigated the DNRA capability and differences between the long filamentous mycelium and short rod-shaped mycelium morphologies of strain EM-B2 under aerobic conditions, using nitrate and nitrite as the sole nitrogen source and glucose as the carbon source, respectively. Fig. 2A demonstrated that the long filamentous mycelial morphology of strain EM-B2 completely consumed the nitrate within 144 h of cultivation. Furthermore, ammonium production gradually increased over the cultivation period, reaching the peak concentration of 44.78 mg/L at 216 h. The production efficiency of ammonium accounts for 46.61% of the converted nitrate. Hydroxylamine and nitrite accumulation were not detected throughout the incubation period, suggesting that its DNRA pathway likely proceeded via NO_3_^-^-N→NH_4_^+^-N. In contrast, as previously reported by Wang et al. (13), the short rod-shaped mycelium of strain EM-B2 retained a residual nitrate concentration of 17.65 mg/L even after 144 h of cultivation (Fig. 2B). The initial inference was that the incomplete conversion of nitrate might be attributed to the depletion of certain nutrients in the medium. Notably, the ammonium concentration rapidly increased to 55.10 mg/L at 144 h. The ammonium content peaked at 63.61 mg/L, with the cultivation period to 216 h, which was significantly higher than that of most of reported microorganisms, such as *Bacillus paralicheniformis* LMG 6934 (17.82 mg/L) Sun et al. (20) and *Pseudomonas putida* Y-9 (3.42 mg/L and 6.66 mg/L) (8), although it was slightly lower than that of the anaerobic strain *Desulfovibrio* sp. CMX (69.63 mg/L) (7). Furthermore, the aerobic DNRA process was accompanied by the generation of a trace amount of nitrite (0.46 mg/L), and hydroxylamine accumulation remained undetectable throughout the incubation period. Therefore, it was speculated that the DNRA pathway of the short rod-shaped mycelium of strain EM-B2 might be NO_3_^-^-N→NO_2_^-^-N→NH_4_^+^-N.

The aforementioned experiments demonstrated that both morphological forms of strain EM-B2 could efficiently produce ammonium in nitrate-amended synthetic wastewater. Specifically, the long filamentous mycelium exhibited superior nitrate conversion capability, whereas the short rod-shaped mycelium achieved higher efficiency in converting the nitrate to ammonium, which indicated a more efficient DNRA process. Currently, the mechanism underlying this divergent nitrogen metabolism functionality within the same strain due to morphological differences remained unclear and might be associated with genetic regulation. It is worth noting that this study does not resolve whether the two observed morphotypes share an identical genome. Although they originated from the same primary isolate, the possibility of spontaneous mutations or genetic divergence during preservation cannot be excluded. Consequently, the functional differences reported here (DNRA capacity and nitrate tolerance) should be interpreted as morphotype-dependent rather than being conclusively attributed to phenotypic plasticity of a single genotype. To address this issue, whole-genome sequencing and comparative genomic analysis are currently underway to distinguish between phenotypic variation and genetic mutation. Regardless of the genetic basis, the key conclusion of this study that morphological state correlates strongly with aerobic DNRA performance remains valid.

#### DNRA with nitrite as the nitrogen source

Nitrite is a common toxic environmental pollutant and a crucial intermediate product in the DNRA process. Currently, only a limited number of microbial strains are known to perform the dissimilatory nitrite reduction to ammonium under aerobic conditions. Therefore, it is significant to investigate the DNRA process in different morphological forms of strain EM-B2 using nitrite as the nitrogen source. Such investigation holds important implications for discovering microbial strains with high efficiency dissimilatory nitrite reduction to ammonium capability and for improving nitrogen resource recovery efficiency.

**Fig 3:**
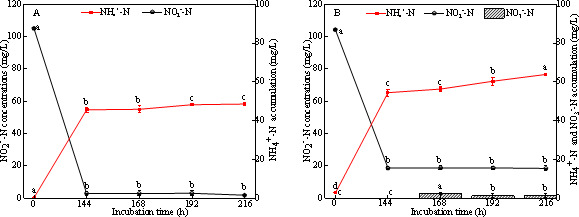
Effects of EM-B2 strains with different morphologies on DNRA using NO_2_^-^-N as nitrogen source. (**A**) The NO_2_^-^-N transformation and NH_4_^+^-N accumulation performances of the long filamentous mycelium strain EM-B2. (**B**) The NO_2_^-^-N transformation, NH_4_^+^-N, and NO_3_^-^-N accumulation performances of the short rod-shaped mycelium strain EM-B2. Values are means ± SD for three replicates. Different letters indicate significant differences between treatments at *P* < 0.05.

Figure 3A showed that when nitrite served as the nitrogen source, the short rod-shaped mycelium morphology of strain EM-B2 rapidly reduced nitrite and produced ammonium within 144 h, achieving an ammonium accumulation of 45.61 mg/L and a nitrite conversion efficiency of 97.06%. In contrast to typical DNRA microorganisms like Anaeromyxobacter, which could only reduce nitrate to ammonium (21), strain EM-B2 was capable of utilizing both nitrate and nitrite for ammonium production, further highlighting its unique status in the nitrogen cycle. Concurrently, most reported strains, such as *Escherichia coli* (9) and *Desulfovibrio* sp. CMX (7), required the addition of specific substances (such as ascorbic acid and iron) to convert nitrite to ammonium, which increased operational costs or risks causing secondary pollution. Furthermore, no nitrate accumulation was detected throughout the DNRA process using nitrite as the nitrogen source in the long filamentous mycelium of EM-B2. The results indicated the absence of a concurrent nitrite oxidation pathway. Consequently, the shortcut pathway involving the direct reduction of nitrite to ammonium ensured both the high conversion efficiency of nitrite and the rapid production of ammonium.

In contrast to the long filamentous mycelium, the short rod-shaped mycelium morphology of strain EM-B2 exhibited a lower nitrite conversion efficiency of merely 82.20% (Fig. 3B). However, a high ammonium yield was achieved at 54.40 mg/L at 144 h, accompanied by minor nitrate accumulation. This observation suggested the concurrent presence of a nitrite oxidation pathway alongside the DNRA process in this strain. This divergence might be correlated with differences in nitrate reductase activity or variations in substrate uptake capacity stemming from the distinct morphological structures of the morphologies. Future research should integrate gene expression profiling and enzyme activity assays to further elucidate these mechanisms. Additionally, existing studies have confirmed that nitrite addition exerted an inhibitory effect on the DNRA process, as observed in strains such as *Pichia kudriavzevii* XTY1, *Roseovarius lacus* sp. nov. GSS12T, and *Enterobacter asburiae* GS2 (22, 23). Conversely, nitrite exhibited no biological inhibition or disruption of nitrogen metabolism toward either the long filamentous mycelium and short rod-shaped mycelium morphologies of strain EM-B2. Meanwhile, this strain EM-B2 efficiently converted nitrite, underscoring its ecological advantage. In addition, some studies suggested that denitrification dominates when nitrite served as the terminal electron acceptor (24). However, our findings contradicted this paradigm. This discrepancy might be associated with microbial species specificity and genetic regulation. In summary, nitrite imposed no biological inhibition on this strain EM-B2, and both morphological forms of EM-B2 efficiently performed DNRA using nitrite as the nitrogen source. Although the short rod-shaped mycelium morphology exhibited a lower nitrite conversion efficiency compared to the long filamentous mycelium, it achieved superior ammonium production, indicating that the short rod-shaped mycelium of strain EM-B2 reduced a larger proportion of nitrite to ammonium. Therefore, short rod-shaped mycelium of strain EM-B2 was selected for subsequent investigation.

### Analysis of DNRA capacity at low concentrations of nitrate/nitrite

Laverman et al. (25) reported that DNRA was dominant under low-concentration nitrate conditions, suggesting that DNRA might be the primary nitrogen conversion pathway in specific environments. Although the short rod-shaped mycelium strain EM-B2 could carry out the DNRA process in wastewater with high concentrations of nitrate or nitrite, when glucose was used as the carbon source and an initial nitrogen source of 100 mg/L, approximately 18 mg/L of nitrate or nitrite nitrogen remained consistently. Therefore, this study aimed to clarify why strain EM-B2 could not completely convert nitrate and nitrite. It also aimed to verify the conversion ability of the strain toward a low-concentration nitrogen source (20 mg/L). To achieve this, experiments were performed using 20 mg/L nitrate or nitrite as the sole nitrogen source.

**Fig 4:**
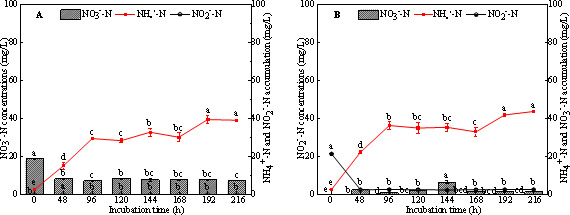
The DNRA capacity of short rod-shaped mycelium strain EM-B2 under sole low-concentration NO_3_^-^-N and NO_2_^-^-N conditions. (**A**) The NO_3_^-^-N transformation, NH_4_^+^-N and NO_2_^-^-N accumulation performances with NO_3_^-^-N as a nitrogen source. (**B**) The NO_2_^-^-N transformation, NH_4_^+^-N, and NO_3_^-^-N accumulation performances with NO_2_^-^-N as a nitrogen source. Values are means ± SD for three replicates. Different letters indicate significant differences between treatments at *P* < 0.05.

As shown in Fig. 4A, when 20 mg/L of nitrate served as the sole nitrogen source, only 56.14% of the nitrate was converted after 48 h of cultivation, with concomitant generation of 0.02 mg/L nitrite. Upon extending the incubation period to 96 h, the intermediate nitrite product became undetectable, while the ammonium concentration progressively increased to 29.35 mg/L. Continued cultivation until 192 h resulted in a peak ammonium yield of 39.33 mg/L. Notably, the ammonium yield substantially exceeded the initial nitrate input, indicating concurrent ammonium generation via mineralization during the DNRA process. Furthermore, despite prolonging cultivation to 216 h, the residual nitrate concentration remained persistently stable at approximately 7 mg/L. These results suggested that the incomplete nitrate removal was not primarily due to nutrient exhaustion, but rather reflected an inherent physiological limitation of strain EM-B2 to reduce nitrate below a certain threshold under the tested conditions. This phenomenon was consistent with previous reports that DNRA bacteria often exhibited a kinetic threshold for nitrate utilization, below which further reduction became negligible (26). From a bioenergetic perspective, under nitrate-limited conditions, the trade-off between energy conservation and substrate affinity could result in a residual nitrate concentration that could not be further reduced (Ghyoot & Gypens, 2022).

Under identical conditions and procedures, the DNRA capability of strain EM-B2 under low-concentration nitrite conditions was further investigated (Fig. 4B). Similar to the low-concentration nitrate scenario, strain EM-B2 rapidly produced ammonium within 0–96 h, with the ammonium concentration increasing from 2.62 to 36.18 mg/L. A nitrite conversion efficiency of 88.88% was achieved by 48 h, after which the residual nitrite stabilized at approximately 2.52 mg/L. Although low-concentration nitrite did not impede the growth of strain EM-B2, it might specifically inhibit the nitrite reductase activity or other associated nitrogen metabolic activities of this strain through mechanisms such as genetic regulation, thereby resulting in incomplete inorganic nitrogen conversion (27). Notably, compared to nitrate as the sole nitrogen source, strain EM-B2 exhibited higher conversion efficiency and lower residual initial nitrogen when utilizing nitrite. After 192 h of cultivation, ammonium production (41.77 mg/L) significantly surpassed that observed with nitrate. These findings indicated that nitrite facilitated the DNRA process more effectively than nitrate. Additionally, analogous to observations with high-concentration nitrite, trace amounts of nitrate were generated during nitrite reduction, likely due to the coexistence of a nitrite oxidation pathway. In summary, strain EM-B2 efficiently performed DNRA even in low-concentration nitrate or nitrite environments, offering a flexible solution for sustainable nitrogen utilization and recovery in low-strength wastewater.

### Analysis of DNRA capacity at high concentrations of nitrite

Previous research indicated that nitrite exerted an inhibitory effect on DNRA efficiency (22). However, preliminary investigations revealed that strain EM-B2 efficiently utilized nitrite for ammonium production. Nevertheless, its capability to reduce high-concentration nitrite to ammonium remained undetermined. Therefore, nitrite concentrations were set at 100, 200, 300, 400, and 500 mg/L to explore the ammonium production capacity of strain EM-B2, respectively.

**Fig 5:**
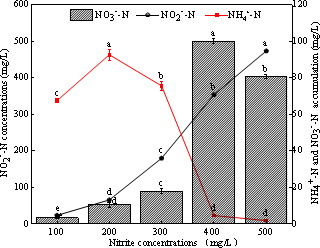
The DNRA capacity of short rod-shaped mycelium strain EM-B2 under conditions of high NO_2_^-^-N concentration. Values are means ± SD for three replicates. Different letters indicate significant differences between treatments at *P* < 0.05.

As depicted in Fig. 5, the ammonium production by strain EM-B2 increased progressively with rising nitrite concentrations, reaching a peak yield of 92.46 mg/L at 200 mg/L nitrite, corresponding to a nitrite conversion efficiency of 88.60%. Concurrently, 10.54 mg/L of nitrate was generated. The ability of strain EM-B2 to perform DNRA under high nitrite concentrations was similar to that observed in *Geobacter sulfurreducens* (28). This contrasted with the findings of Amo & Bañeras (22), who reported that nitrite inhibited the DNRA process in their specific strain. This phenomenon indicated a unique advantage of strain EM-B2 for treating high-concentration nitrite wastewater. However, when the nitrite concentration reached 300 mg/L, ammonium production gradually decreased to 75.46 mg/L. At 400 mg/L nitrite, the ammonium concentration plummeted sharply to 4.65 mg/L, presumably attributable to the inhibition of nitrogen metabolism functions resulting from excessively high nitrite concentrations exceeding the toxicity threshold. These findings aligned with observations in *Leclercia adecarboxylata* strain AS3-1 and *Rhodotorula diobovata* DSBCA06 (29, 30), where elevated nitrite concentrations similarly suppress microbial growth and nitrogen conversion efficiency. In summary, strain EM-B2 demonstrated the capacity to tolerate moderately high nitrite concentrations while effectively reducing them to ammonium. This conversion process contributed to maintaining aquatic nitrogen equilibrium and supplied bioavailable nitrogen for plant uptake, thereby enhancing ecosystem health and stability. Concurrently, it offered viable solutions for addressing nitrogen pollution in food processing, synthetic leather wastewater, and agricultural systems.

### The specific activity determination of nitrite reductase

The transformation of inorganic nitrogen by environmental microorganisms largely relies on enzymatic catalysis. The key enzyme in the DNRA process is nitrite reductase (NIR) (31). To comprehensively characterize the DNRA nitrogen metabolism of strain EM-B2, the specific activity of its NIR was determined. The results confirmed the successful detection of NIR in strain EM-B2, with a specific activity of 0.024 U/mg protein, indicating NIR’s involvement in the aerobic DNRA process of this strain. Previous studies have established that oxygen impeded nitrate and nitrite reduction. This inhibitory effect primarily occurred by suppressing the synthesis and activity of key reductases and blocking the nitrate transmembrane transport system, ultimately preventing efficient nitrate reduction (32, 33). Remarkably, strain EM-B2 efficiently performed DNRA via NIR even under aerobic conditions. Furthermore, the specific activity of NIR in strain EM-B2 was significantly higher than that in *P. putida* Y-9 (0.006 U/mg protein) (28) and *Acinetobacter* sp. Y1 (0.008 ± 0.0003 U/mg protein) (34). It was comparable to that in *Alcaligenes faecalis* C16 (0.021 U/mg protein) (35), *Klebsiella pneumoniae* CF-S9 (0.02 U/mg protein) (36), and *Diaphorobacter* sp. PD-7 (0.019 ± 0.005 U/mg protein) (37) but significantly lower than that in *P. taiwanensis* J488 (0.10 U/mg protein) (31). The successful detection of NIR activity confirmed the presence and catalytic function of this enzyme in EM-B2 during the DNRA process.

### ^15^N isotope tracer analysis

A ¹⁵N isotope experiment was performed to further verify the nitrogen metabolism pathway of strain EM-B2. This was done to determine whether the pathway was DNRA. All results are shown in Table 1. After 216 h of aerobic cultivation in the medium containing ¹⁵NO₃⁻, the initial ¹⁵NO₃⁻ concentration decreased from 93.35 mg/L to 2.50 mg/L. The initial ¹⁵NO₃⁻ abundance was 9.69%. Concurrently, substantial accumulation of extracellular NH₄^+^ (63.71 mg/L) was detected, with a ¹⁵N abundance of 7.11 atom%. In contrast, the intracellular NH₄^+^ exhibited a ¹⁵N abundance of only 0.05 atom%, showing no marked difference from the initial level. This indicated the absence of significant assimilation of the ¹⁵N-labeled nitrogen source within the cells. Collectively, strain EM-B2 converted the majority of ¹⁵NO₃⁻ into extracellular ¹⁵NH₄^+^, confirming DNRA as the primary source of NH₄^+^ in the supernatant. In addition, in batch continuous experiments using nitrate as the sole nitrogen source, ammonium increased gradually along with the decrease in nitrate during this process. Therefore, the ¹⁵N-tracing result at 216 h served as confirmatory endpoint evidence, while the dynamic trajectory was provided by the unlabeled batch data. This phenomenon further confirmed that the production of ammonium was derived from the aerobic DNRA process of strain EM-B2. While DNRA typically occurred only under anaerobic conditions (38), this study was conducted under aerobic conditions, demonstrating the efficient occurrence of the DNRA process in the presence of oxygen.

**TABLE 1:** Isotopic analysis of Na¹⁵NO₃ in *Streptomyces mediolani* EM-B2

Time		NO3—N		NH_4_^+^-N	
		**^15^N isotopic ratios%**	**Content (mg/L**)	**^15^N isotopic ratios%**	**Content (mg/L**)
0 h	Extracellular	9.69 ± 0.07	93.35 ± 0.60	0.49 ± 0.00	2.44 ± 0.16
	Intracellular	0.10 ± 0.13	1.55 ± 0.60	0.07 ± 0.02	0.49 ± 0.41
216 h	Extracellular	0.53 ± 0.02	2.50 ± 0.21	7.11 ± 0.01	63.71 ± 0.70
	Intracellular	0.00	2.30 ± 0.55	0.05 ± 0.04	0.67 ± 0.08

### Conclusions

This study provided significant insights into the efficiency and pathways of DNRA in actinobacteria under aerobic conditions. *S. mediolani* EM-B2 was an aerobic high ammonium-producing strain. This actinomycete exhibited two distinct morphologies (long filamentous mycelium and short rod-shaped mycelia). Both mycelial morphologies efficiently reduced nitrate and nitrite to ammonium under aerobic conditions. The short rod-shaped mycelium of strain EM-B2 exhibited a superior DNRA capability compared to the long filamentous mycelium, which was associated with divergent nitrogen metabolic pathways. Compared with nitrate, strain EM-B2 was more conducive to aerobic DNRA using nitrite and could tolerate up to 300 mg/L of nitrite. Enzyme activity assays and ¹⁵N isotopic tracing confirmed DNRA functionality of strain EM-B2 in the short rod-shaped mycelia, with a key NIR-specific activity of 0.024 U/mg protein. These findings indicated a DNRA pathway likely proceeding via NO₃⁻ → NO₂⁻ → NH₄^+^.

## Supplementary Material

Reviewer comments

## Data Availability

The original contributions presented in the study are included in the article; further inquiries can be directed to the corresponding authors.

## SUPPLEMENTAL MATERIAL

OPEN PEER REVIEW reviewer-comments.pdf An accounting of the reviewer comments and feedback.

ASM does not own the copyrights to Supplemental Material that may be linked to, or accessed through, an article. The authors have granted ASM a non-exclusive, world-wide license to publish the Supplemental Material files. Please contact the corresponding author directly for reuse.
