# Supplementary material for: Mycelial morphology influenced aerobic DNRA in Streptomyces mediolani EM-B2: short rod-shaped mycelium showed markedly greater efficiency than long filamentous mycelium
Source: Microbiol Spectr. 2026 Jul 2;14(8):e00709-26. doi: 10.1128/spectrum.00709-26 (PMC13435786; doi:10.1128/spectrum.00709-26)
Supplement: Supplementary file 1 [file reviewer-comments.pdf]

AMERICAN SOCIETY FOR MICROBIOLOGY | Microbiology Spectrum

Mycelial morphology influenced aerobic DNRA in *Streptomyces mediolani* EM-B2: short rod-shaped mycelium showed markedly greater efficiency than long filamentous mycelium

Manman Zhang, Tengxia He, Cerong Wang, Yixiao Liao, Hong Zhao, and chengtao Jin

Corresponding Author(s): Tengxia He, Guizhou University

Review Timeline:

Submission Date:	February 26, 2026
Editorial Decision:	April 15, 2026
Revision Received:	May 6, 2026
Accepted:	May 13, 2026

Editor: Jing Han

Reviewer(s): Disclosure of reviewer identity is with reference to reviewer comments included in decision letter(s). The following individuals involved in review of your submission have agreed to reveal their identity: Mengkai Zhou (Reviewer #1)

Transaction Report:

(Note: With the exception of the correction of typographical or spelling errors that could be a source of ambiguity, letters and reports are not edited. The original formatting of letters and referee reports may not be reflected in this compilation.)

DOI: https://doi.org/10.1128/spectrum.00709-26

Re: Spectrum00709-26 (**Mycelial morphology influenced aerobic DNRA in *Streptomyces mediolani* EM-B2: short aerial hypha showed markedly greater efficiency than long branching filaments**)

Dear Prof. Tengxia He:

Thank you for the privilege of reviewing your work. Below you will find my comments, instructions from the Spectrum editorial office, and the reviewer comments.

Please return the manuscript within 60 days; if you cannot complete the modification within this time period, please contact me. If you do not wish to modify the manuscript and prefer to submit it to another journal, notify me immediately so that the manuscript may be formally withdrawn from consideration by Spectrum.

Revision Guidelines
To submit your modified manuscript, log into the submission site at https://spectrum.msubmit.net/cgi-bin/main.plex. Go to Author Tasks and click the appropriate manuscript title to begin. The information you entered when you first submitted the paper will be displayed; update this as necessary. Note the following requirements:

• Upload point-by-point responses to the issues raised by the reviewers in a file named "Response to Reviewers," NOT in your cover letter.
• Upload a compare copy of the manuscript (without figures) as a "Marked-Up Manuscript" file.
• Upload a clean .DOC/.DOCX version of the revised manuscript and remove the previous version.
• Each figure must be uploaded as a separate, editable, high-resolution file (TIFF or EPS preferred), and any multipanel figures must be assembled into one file.
• Any supplemental material intended for posting by ASM should be uploaded with their legends separate from the main manuscript. You can combine all supplemental material into one file (preferred) or split it into a maximum of 10 files with all associated legends included.

For complete guidelines on revision requirements, see our Submission and Review Process webpage. Submission of a paper that does not conform to guidelines may delay acceptance of your manuscript.

Data availability: ASM policy requires that data be available to the public upon online posting of the article, so please verify all links to sequence records, if present, and make sure that each number retrieves the full record of the data. If a new accession number is not linked or a link is broken, provide Spectrum production staff with the correct URL for the record. If the accession numbers for new data are not publicly accessible before the expected online posting of the article, publication may be delayed; please contact production staff (Spectrum@asmusa.org) immediately with the expected release date.

Publication Fees: For information on publication fees and which article types are subject to charges, visit our website. If your manuscript is accepted for publication and any fees apply, you will be contacted separately about payment during the production process; please follow the instructions in that e-mail. Arrangements for payment must be made before your article is published.

ASM Membership: Corresponding authors may join or renew ASM membership to obtain discounts on publication fees. Need to upgrade your membership level? Please contact Customer Service at Service@asmusa.org.

The ASM Journals program strives for constant improvement in our submission and publication process. Please tell us how we can improve your experience by taking this quick Author Survey.

Thank you for submitting your paper to Spectrum.

Sincerely,
Jing Han
Editor
Microbiology Spectrum

Reviewer #1 (Comments for the Author):

1. The manuscript only mentions that the strain exhibits two morphologies (long filamentous mycelium and short rod-shaped aerial hyphae) under the same culture conditions, but fails to specify the specific methods for morphological induction and stable culture, nor verify the morphological stability of the strain.
2. The section of "DNRA medium with nitrite as the nitrogen source" in Section 2.1 contains errors in the writing of chemical

formulas: Fe_2SO_4 in Lines 188 and 190 should be $Fe_2(SO_4)_3$; the expression of "10.0 g/L of tryptone per liter" in Line 181 is incorrect.
3. The abstract states that the proportion of nitrate converted to ammonium by short aerial hyphae is 75.82% (63.63 mg/L), while the value is given as 63.61 mg/L in Section 3.2.1.
4. There is a spelling error in "15N istopic ratios%" in Table 1, with the correct spelling being "isotopic".
5. The legends and axis labels of all figures and tables are non-standard: for example, the letters a/b/c/d in Fig. 2 are not explained.
6. For the 15N isotope tracing experiment, only the results at 0 h and 216 h are presented, with a lack of time-gradient data, which fails to reflect the dynamic changes of 15N conversion from nitrate to ammonium during the DNRA process.

This study found that the aerobic DNRA efficiency of *Streptomyces mediolani* EM-B2 varied with different mycelial morphologies, identified the high ammonium production efficiency and the high nitrite tolerance of short aerial hyphae. It provides a new perspective for the research on actinobacterial DNRA metabolism, and also offers potential microbial resources and technical insights for soil nitrogen retention and nitrogen pollution control. The study design is relatively systematic with sufficient data support, yet the manuscript has numerous issues that need improvement and revision in terms of experimental details and data expression.

1. The manuscript only mentions that the strain exhibits two morphologies (long filamentous mycelium and short rod-shaped aerial hyphae) under the same culture conditions, but fails to specify the specific methods for morphological induction and stable culture, nor verify the morphological stability of the strain.
2. The section of "DNRA medium with nitrite as the nitrogen source" in Section 2.1 contains errors in the writing of chemical formulas: Fe_2SO_4 in Lines 188 and 190 should be $Fe_2(SO_4)_3$; the expression of "10.0 g/L of tryptone per liter" in Line 181 is incorrect.
3. The abstract states that the proportion of nitrate converted to ammonium by short aerial hyphae is 75.82% (63.63 mg/L), while the value is given as 63.61 mg/L in Section 3.2.1.
4. There is a spelling error in "15N istopic ratios%" in Table 1, with the correct spelling being "isotopic".
5. The legends and axis labels of all figures and tables are non-standard: for

example, the letters a/b/c/d in Fig. 2 are not explained.

6. For the ^{15}N isotope tracing experiment, only the results at 0 h and 216 h are presented, with a lack of time-gradient data, which fails to reflect the dynamic changes of ^{15}N conversion from nitrate to ammonium during the DNRA process.

The manuscript by Zhang et al. investigated the impact of mycelial morphology on DNRA metabolism in *Streptomyces mediolani* EM-B2, a metabolic pathway rarely documented in *Streptomyces* species. The manuscript reports the phenotype but lacks further mechanistic investigation or discussion. And, the language of the manuscript should be rechecked before publication. My comments are as follows:

1, The manuscript mentioned that strain EM-B2 exhibited two distinct morphologies under identical conditions (line 288). It is unclear whether these forms are phenotypic variants or the result of genetic divergence. If the authors got these morphologies from different colonies of EM-B2, there is a risk of genetic heterogeneity or spontaneous mutation. If that's the case, is it still appropriate to characterize these as the same strain?

2, Regarding the residual nitrate observed in the short aerial hyphae (Fig. 2), the authors hypothesized that this was due to the depletion of certain nutrients. Can the authors verify this by adding fresh medium to the culture?

3, Fig. 4, why was there still residual nitrate even under low-concentration of nitrate?

4, Some typos, line 21 "carboon" should be "carbon"; line 24 & 46 "Up date" should be "To date"; Line 121, the most bacteria should be most of the bacteria;

5, Line 142 "airborne mycelium" should be "aerial mycelium"; line 143 "actinted mycelial proteins"? Line 145, *Streptomyces penicillium* should be *S. penicillium*; *Streptomyces mediolani* EM-B2 should be *S. mediolani* EM-B2 when it appeared for the second time? Long filamentous, long filamentous in line 166. Line 210, Different, different. Line 758, istopic should be isotopic

There are too many mistakes in this manuscript and the language of the whole paper should be carefully rechecked.

Dear editor,

The original manuscript number is Spectrum00709-26

Thanks for your review of our manuscript titled "Mycelial morphology influenced aerobic DNRA in *Streptomyces mediolani* EM-B2: short aerial hypha showed markedly greater efficiency than long branching filaments" written by Manman Zhang et al, which was submitted to the journal of ***Microbiology Spectrum***.

Response: Thanks for Editor and Reviewers consideration and evaluation of our manuscript. We have addressed carefully issues point-by-point for Editor individually. The detailed revision has now been added to the manuscript with red color.

Here is the reply to the queries.

Comments from Editors and Reviewers

Reviewers' comments:

#Reviewer 1: This study found that the aerobic DNRA efficiency of *Streptomyces mediolani* EM-B2 varied with different mycelial morphologies, identified the high ammonium production efficiency and the high nitrite tolerance of short aerial hyphae. It provides a new perspective for the research on actinobacterial DNRA metabolism, and also offers potential microbial resources and technical insights for soil nitrogen retention and nitrogen pollution control. The study design is relatively systematic with sufficient data support, yet the manuscript has numerous issues that need improvement and revision in terms of experimental details and data expression.

Response: We sincerely thank the reviewer for the constructive feedback. We have carefully considered the raised critical points and have made corresponding revisions throughout the manuscript to provide the necessary clarifications. All changes have been highlighted in the revised version for the reviewer's convenience. We appreciate the opportunity to further improve our work.

1. The manuscript only mentions that the strain exhibits two morphologies (long filamentous mycelium and short rod-shaped aerial hyphae) under the same culture conditions, but fails to specify the specific methods for morphological induction and stable culture, nor verify the morphological stability of the strain.

Response: We thank the reviewer for this important observation. In our study, the two morphologies (long filamentous mycelium and short rod-shaped mycelium) arise spontaneously during routine cultivation of strain EM-B2 without any additional chemical or physical induction. The short rod-shaped mycelium was derived from the long filamentous mycelium during the growth process. To verify morphological stability, both morphotypes were separately preserved (30% glycerol) and subjected to more than 10 successive subcultures. Under all tested temperature conditions, no morphological reversion was observed. These results confirm that each morphology was stable once established. **The detailed method and stability validation of** "The two morphologies of strain EM-B2, namely long filamentous mycelium and short rod shaped mycelium, arise spontaneously during routine cultivation, with the short rod shaped form deriving from the long filamentous one during cultivation. Both morphotypes were separately preserved and subjected to over 10 successive subcultures. No morphological reversion occurred under any tested temperature, confirming that each form remains stable once established." **have been added to the Materials and Methods**. We apologize for the lack of clarity in the original manuscript.

2. The section of "DNRA medium with nitrite as the nitrogen source" in Section 2.1 contains errors in the writing of chemical formulas: Fe_2SO_4 in Lines 188 and 190 should be $Fe_2(SO_4)_3$; the expression of "10.0 g/L of tryptone per liter" in Line 181 is incorrect.

Response: Thank you very much for your careful review of the manuscript. We have changed the "Fe_2SO_4" in lines 188 and 190 of the manuscript to "$Fe_2(SO_4)_3$", and have also checked the other components of the culture medium. Meanwhile, the word

"per liter" in the sentence "10.0 g/L of tryptone per liter" has been deleted. Once again, we apologize for our negligence.

3. The abstract states that the proportion of nitrate converted to ammonium by short aerial hyphae is 75.82% (63.63 mg/L), while the value is given as 63.61 mg/L in Section 3.2.1.

Response: We thank the reviewer for pointing out this inconsistency. After checking our original experimental data and calculation records, **the correct value is 63.61 mg/L**, corresponding to a nitrate-to-ammonium conversion proportion of 75.82%. The value of 63.63 mg/L in the Abstract was a spelling error during manuscript preparation. We have corrected it to 63.61 mg/L in the revised manuscript. Additionally, we have carefully rechecked all related data in the Abstract and Section 3.2.1, and no other discrepancies were found. We apologize for any confusion caused and thank the reviewer again for the careful reading.

4. There is a spelling error in "15N istopic ratios%" in Table 1, with the correct spelling being "isotopic".

Response: We apologize for this spelling mistake. The correct spelling is "isotopic". We have corrected "15N istopic ratios%" to "^{15}N isotopic ratios%" in Table 1 of the revised manuscript. We have also carefully checked the entire manuscript for any similar typographical errors and confirmed that no other mistakes exist. We thank the reviewer for the careful correction.

5. The legends and axis labels of all figures and tables are non-standard: for example, the letters a/b/c/d in Fig. 2 are not explained.

Response: We thank the reviewer for this valuable suggestion. We agree that all figures and tables should be self-explanatory. Specifically for Fig. 2, the lowercase letters a, b, c, d indicated statistically significant differences between groups ($p < 0.05$) based on One-way ANOVA test of IBM SPSS Statistics software. We have now clearly explained this in the revised all figures caption as follows: "Values are means

± SD for three replicates. Different letters indicate significant differences between treatments at $p < 0.05$". Additionally, The inconsistency in the coordinate labels in all the figures was due to the fact that the figures did not show the substances that did not accumulate. For example, in Fig. 2A, when the strain EM-B2 of long branching filaments used nitrate as the sole nitrogen source, only ammonium nitrogen accumulate, with no nitrite detected. Consequently, the right vertical axis of this figure did not display nitrite concentration. In contrast, when the EM-B2 strain of short rod-shaped mycelium applied nitrate as the sole nitrogen source (Fig. 2B), both ammonium and nitrite were produced; therefore, the right vertical axis presented the concentrations of both substances. The same rationale explains the inconsistencies in axis labeling observed in the other figures. **In order to make the meaning of the chart clearer, the annotation content in the Fig. 2 section has been changed from** "Fig. 2 Effects of EM-B2 strains with different morphologies on DNRA using nitrate as nitrogen source. A, long branching filaments; B, short aerial hypha" **to** "**Fig. 2** Effects of EM-B2 strains with different morphologies on DNRA using NO_3^--N as nitrogen source. A, The NO_3^--N transformation and NH_4^+-N accumulation performances of the long filamentous mycelium strain EM-B2; B, The NO_3^--N transformation, NH_4^+-N and NO_2^--N accumulation performances of the short rod-shaped mycelium strain EM-B2. Values are means ± SD for three replicates. Different letters indicate significant differences between treatments at $p < 0.05$"; In Fig. 3, **the content of** "Fig. 3 Effects of EM-B2 strains with different morphologies on DNRA using nitrite as nitrogen source. A, long branching filaments; B, short aerial hypha" **has been advised as** "**Fig. 3** Effects of EM-B2 strains with different morphologies on DNRA using NO_2^--N as nitrogen source. A, The NO_2^--N transformation and NH_4^+-N accumulation performances of the long filamentous mycelium strain EM-B2; B, The NO_2^--N transformation, NH_4^+-N and NO_3^--N accumulation performances of the short rod-shaped mycelium strain EM-B2. Values are means ± SD for three replicates. Different letters indicate significant differences between treatments at $p < 0.05$"; Similarly, in Fig. 4 and 5, **the contents of** "Fig. 4 The DNRA capacity under low-concentration nitrate and nitrite conditions. A, nitrate;

B, nitrite" **and** "Fig. 5 The DNRA capacity under conditions of high concentration of nitrite" **has also been as** "**Fig. 4** The DNRA capacity of short rod-shaped mycelium strain EM-B2 under sole low-concentration NO_3^--N and NO_2^--N conditions. A, The NO_3^--N transformation, NH_4^+-N and NO_2^--N accumulation performances with NO_3^--N as a nitrogen source; B, The NO_2^--N transformation, NH_4^+-N and NO_3^--N accumulation performances with NO_2^--N as a nitrogen source. Values are means ± SD for three replicates. Different letters indicate significant differences between treatments at $p < 0.05$" and "**Fig. 5** The DNRA capacity of short rod-shaped mycelium strain EM-B2 under conditions of high NO_2^--N concentration. Values are means ± SD for three replicates. Different letters indicate significant differences between treatments at $p < 0.05$".

6. For the 15N isotope tracing experiment, only the results at 0 h and 216 h are presented, with a lack of time-gradient data, which fails to reflect the dynamic changes of 15N conversion from nitrate to ammonium during the DNRA process.

Response: We thank the reviewer for this constructive comment. We agree that time-gradient ^{15}N isotope data would better reveal the dynamic conversion from nitrate to ammonium via DNRA. However, due to the high cost and limited access to ^{15}N-labeled substrates, we were able to perform the isotope tracing only at the start (0 h) and at the endpoint (216 h), which was selected based on preliminary experiments showing near-complete nitrate consumption and stabilized ammonium accumulation by this time. Importantly, the dynamic process of nitrate-to-ammonium conversion was already captured in our parallel unlabeled batch experiments (48 h interval detection). Therefore, the ^{15}N tracing result at 216 h served as a confirmatory endpoint evidence, while the dynamic trajectory was provided by the unlabeled batch data. We have clarified this in the revised manuscript. In the part of **"3.6 ^{15}N isotope tracer analysis"**, the content of "In addition, in batch continuous experiments using nitrate as the sole nitrogen source, ammonium increased gradually along with the decrease of nitrate during this process. Therefore, the ^{15}N tracing result at 216 h served as a confirmatory endpoint evidence, while the dynamic trajectory was

provided by the unlabeled batch data. This phenomenon further confirmed that the production of ammonium was derived from the aerobic DNRA process of strain EM-B2. " was added.

#Reviewer 2

The manuscript by Zhang et al. investigated the impact of mycelial morphology on DNRA metabolism in *Streptomyces mediolani* EM-B2, a metabolic pathway rarely documented in *Streptomyces* species. The manuscript reports the phenotype but lacks further mechanistic investigation or discussion. And, the language of the manuscript should be rechecked before publication. My comments are as follows,

Response: We sincerely thank the reviewer for the constructive feedback. We have carefully considered the raised critical points and have made corresponding revisions throughout the manuscript to provide the necessary clarifications. All changes have been highlighted in the revised version for the reviewer's convenience. Meanwhile, the language and grammar of this manuscript was rechecked and revised by Lisa Oberding, MSc, from Liwen Bianji (Edanz) (www.liwenbianji.cn). We appreciate the opportunity to further improve our work.

1. The manuscript mentioned that strain EM-B2 exhibited two distinct morphologies under identical conditions (line 288). It is unclear whether these forms are phenotypic variants or the result of genetic divergence. If the authors got these morphologies from different colonies of EM-B2, there is a risk of genetic heterogeneity or spontaneous mutation. If that's the case, is it still appropriate to characterize these as the same strain?

Response: We thank the reviewer for raising this critical point regarding the genetic basis of the two morphologies. In the present study, the two morphologies (long filamentous mycelium and short rod-shaped mycelium) arise spontaneously during routine cultivation of strain EM-B2 without any additional chemical or physical induction. The short rod-shaped mycelium was derived from the long filamentous mycelium during the growth process. To verify morphological stability,

both morphotypes were separately preserved (30% glycerol) and subjected to more than 10 successive subcultures. Under all tested temperature conditions, no morphological reversion was observed. These results confirm that each morphology was stable once established. We fully agree with the reviewer that we cannot currently exclude the possibility of spontaneous mutation or genetic divergence between the two morphotypes, as whole-genome sequencing of two morphologies has not yet been performed. **However, we wish to clarify that the scope of this manuscript was phenotypic and functional characterization of the two stable morphotypes, not genetic taxonomy.** The key findings are: (1) Two stable, heritable morphotypes existed under identical culture conditions after preservation; (2) They exhibited significantly different aerobic DNRA capacities, with the short rod-shaped mycelium showing stronger activity and tolerance to high nitrate (500 mg/L); (3) These differences were reproducible across multiple passages (>10 transfers). Whether the two morphotypes represent phenotypic plasticity or genetic divergence does not affect the validity of the observed functional differences (both scenarios still support the conclusion that morphological state correlates with DNRA performance). Of course, in our subsequent research, we will continue to explore at the molecular level (whole-genome sequencing and comparative genomic analysis) the reasons for the morphological differentiation of the bacterial strains.

To be scientifically rigorous, we have added the following in the revised manuscript. Fristly, a clear statement that genetic homogeneity has not been confirmed. **the content** "However, whether these two morphotypes arise from phenotypic plasticity (same genotype) or spontaneous genetic mutation (different genotypes) remains to be determined. In the present study, we therefore refer to them as two stable morphotypes derived from the same original isolate, rather than asserting genetic identity. Therefore, it is necessary to conduct further research on the DNRA ability of this strain EM-B2 at the metabolic and molecular levels in different forms." **was added to the part of "3.1 The different mycelial morphologies of strain EM-B2**"; Secondly, A change in terminology from "the same strain" to "two stable morphotypes derived from the same original isolate" to avoid over-claim. **The**

content of "During routine cultivation, strain EM-B2 spontaneously gave rise to two stable morphologies（long filamentous mycelium and short rod-shaped mycelium, with the latter originating from the former during growth. Both morphotypes remained stable after more than 10 successive subcultures under all tested temperatures, with no reversion observed. However, the aerobic DNRA capabilities of two stable morphotypes derived from the same original isolate strain EM-B2 are unclear." **was added to 3.1 The different mycelial morphologies of strain EM-B2**. Thirdly, A brief statement "It is worth noting that this study does not resolve whether the two observed morphotypes share an identical genome. Although they originated from the same primary isolate, the possibility of spontaneous mutations or genetic divergence during preservation cannot be excluded. Consequently, the functional differences reported here (DNRA capacity, nitrate tolerance) should be interpreted as morphotype-dependent rather than being conclusively attributed to phenotypic plasticity of a single genotype. To address this issue, whole-genome sequencing and comparative genomic analysis are currently underway to distinguish between phenotypic variation and genetic mutation. Regardless of the genetic basis, the key conclusion of this study that morphological state correlates strongly with aerobic DNRA performance remains valid." in the **3.2.1 DNRA with nitrate as the nitrogen source** that future work is needed to distinguish phenotypic plasticity from genetic mutation.

2. Regarding the residual nitrate observed in the short aerial hyphae (Fig. 2), the authors hypothesized that this was due to the depletion of certain nutrients. Can the authors verify this by adding fresh medium to the culture?

Response: We thank the reviewer for this insightful suggestion. In the DNRA optimization experiment with nitrate as the nitrogen source using strain EM-B2, previously reported by Wang et al. (2025), nitrate was not completely converted even when the C/N ratio was raised to 25. This suggested that nutrient depletion was not the primary cause. In addition, to test whether the residual nitrate (18 mg/L) at 216 h in the high nitrate culture (initial 100 mg/L) was caused by nutrient depletion, we

performed an additional experiment using a lower initial nitrate concentration (20 mg/L) in fresh medium, while keeping all other nutrients the same **(3.3 Analysis of DNRA capacity at low concentrations of nitrate/nitrite).** If nutrient depletion were the main cause, we would expect the low nitrate culture to achieve complete removal. However, even after extending the incubation to 216 h, the residual nitrate stabilized at approximately 7 mg/L (Fig. 4). This residual level was proportionally similar to that observed in the high nitrate experiment (18 mg/L residual from an initial higher concentration). These results suggested that the incomplete nitrate removal was not primarily due to nutrient exhaustion, but rather reflected an inherent physiological limitation of this strain EM-B2 to reduce nitrate below a certain threshold under the tested conditions. **Therefore, in order to more clearly explain the reason why strain EM-B2 could not completely convert nitrate, we added the content of** "These results suggested that the incomplete nitrate removal was not primarily due to nutrient exhaustion, but rather reflected an inherent physiological limitation of strain EM-B2 to reduce nitrate below a certain threshold under the tested conditions. This phenomenon was consistent with previous reports that DNRA bacteria often exhibited a kinetic threshold for nitrate utilization, below which further reduction became negligible (Sorokin et al., 2023). From a bioenergetic perspective, under nitrate-limited conditions, the trade-off between energy conservation and substrate affinity could result in a residual nitrate concentration that could not be further reduced (Ghyoot & Gypens, 2022)." in the section "**3.3 Analysis of DNRA capacity at low concentrations of nitrate/nitrite**".

3. Fig. 4, why was there still residual nitrate even under low-concentration of nitrate?

Response: We thank the reviewer for this insightful suggestion. In the DNRA optimization experiment with nitrate as the nitrogen source using strain EM-B2, previously reported by Wang et al. (2025), nitrate was not completely converted even when the C/N ratio was raised to 25. This suggested that nutrient depletion was not the primary cause. In addition, to test whether the residual nitrate (18 mg/L) at 216 h in the high nitrate culture (initial 100 mg/L) was caused by nutrient depletion, we

performed an additional experiment using a lower initial nitrate concentration (20 mg/L) in fresh medium, while keeping all other nutrients the same **(3.3 Analysis of DNRA capacity at low concentrations of nitrate/nitrite).** If nutrient depletion were the main cause, we would expect the low nitrate culture to achieve complete removal. However, even after extending the incubation to 216 h, the residual nitrate stabilized at approximately 7 mg/L (Fig. 4). This residual level was proportionally similar to that observed in the high nitrate experiment (18 mg/L residual from an initial higher concentration). These results suggested that the incomplete nitrate removal was not primarily due to nutrient exhaustion, but rather reflected an inherent physiological limitation of this strain EM-B2 to reduce nitrate below a certain threshold under the tested conditions. **Therefore, in order to more clearly explain the reason why strain EM-B2 could not completely convert nitrate, we added the content of** "These results suggested that the incomplete nitrate removal was not primarily due to nutrient exhaustion, but rather reflected an inherent physiological limitation of strain EM-B2 to reduce nitrate below a certain threshold under the tested conditions. This phenomenon was consistent with previous reports that DNRA bacteria often exhibited a kinetic threshold for nitrate utilization, below which further reduction became negligible (Sorokin et al., 2023). From a bioenergetic perspective, under nitrate-limited conditions, the trade-off between energy conservation and substrate affinity could result in a residual nitrate concentration that could not be further reduced (Ghyoot & Gypens, 2022)." in the section "**3.3 Analysis of DNRA capacity at low concentrations of nitrate/nitrite**".

4. Some typos, line 21 "carboon" should be "carbon"; line 24 & 46 "Up date" should be "To date"; Line 121, the most bacteria should be most of the bacteria;

Response: We thank the reviewer for carefully checking the manuscript and pointing out these typographical and grammatical errors. We have corrected them as follows: Line 21: "carboon" has been corrected to "carbon". Line 24 and Line 46: "Up date" has been corrected to "To date". Line 121: "the most bacteria" has been corrected to "most of the bacteria". These corrections have been marked in the revised

manuscript. We apologize for these oversights and thank the reviewer again for their meticulous reading.

5. Line 142 "airborne mycelium" should be "aerial mycelium"; line 143 "actinted mycelial proteins"? Line 145, *Streptomyces penicillium* should be *S. penicillium*; *Streptomyces mediolani* EM-B2 should be *S. mediolani* EM-B2 when it appeared for the second time? Long filamentous, long filamentous in line 166. Line 210, Different, different. Line 758, istopic should be isotopic.

Response: We thank the reviewer for their careful reading and for identifying these typographical and formatting errors. The “airborne mycelium” has been corrected to “aerial mycelium”; The “actinted mycelial proteins” was revised as “actinorhodin”; The species “*Streptomyces penicillium*” appeared only once in the article, so it was not abbreviated in the manuscript. “*Streptomyces mediolani* EM-B2” has been corrected to “*S. mediolani* EM-B2” for its second appearance in abstract and main text.

We have checked all of the problems, and modified all errors. Thanks for your consideration of our manuscript again.

Yours sincerely,

Tengxia He, professor,

Corresponding author Address: College of Life Sciences, Guizhou University, Huaxi, Guiyang, Guizhou 550025, China.

Tel.: 0851-88292178;

Fax: 0851-83621956.

E-mail address: txhe@gzu.edu.cn (T.X. He).

Re: Spectrum00709-26R1 (**Mycelial morphology influenced aerobic DNRA in *Streptomyces mediolani* EM-B2: short rod-shaped mycelium showed markedly greater efficiency than long filamentous mycelium**)

Dear Prof. Tengxia He:

Your manuscript has been accepted, and I am forwarding it to the ASM production staff for publication. Your paper will first be checked to make sure all elements meet the technical requirements. ASM staff will contact you if anything needs to be revised before copyediting and production can begin. Otherwise, you will be notified when your proofs are ready to be viewed.

Data Availability: ASM policy requires that data be available to the public upon online posting of the article, so please verify all links to sequence records, if present, and make sure that each number retrieves the full record of the data. If a new accession number is not linked or a link is broken, provide production staff with the correct URL for the record. If the accession numbers for new data are not publicly accessible before the expected online posting of the article, publication may be delayed; please contact ASM production staff immediately with the expected release date.

Publication Fees: For information on publication fees and which article types have charges, please visit our website. We have partnered with Copyright Clearance Center (CCC) to collect author charges. If fees apply to your paper, you will receive a message from no-reply@copyright.com with further instructions. For questions related to paying charges through RightsLink, please contact CCC at ASM_Support@copyright.com or toll free at +1-877-622-5543. CCC makes every attempt to respond to all emails within 24 hours.

ASM Membership: Corresponding authors may join or renew ASM membership to obtain discounts on publication fees. Need to upgrade your membership level? Please contact Customer Service at Service@asmusa.org.

Embargo Policy: A press release may be issued as soon as the manuscript is posted on the Spectrum Latest Articles webpage. The corresponding author will receive an email with the subject line "ASM Journals Author Services Notification" when the article is available online.

The ASM Journals program strives for constant improvement in our submission and publication process. Please tell us how we can improve your experience by taking this quick Author Survey.

Thank you for submitting your paper to Spectrum.

Sincerely,
Jing Han
Editor
Microbiology Spectrum

Reviewer #2 (Comments for the Author):

I have no further concerns.
